# Neural Signatures of Auditory Perceptual Bistability Revealed by Large-Scale Human Intracranial Recordings

**DOI:** 10.1523/JNEUROSCI.0655-18.2019

**Published:** 2019-08-14

**Authors:** Rodica Curtu, Xiayi Wang, Bingni W. Brunton, Kirill V. Nourski

**Affiliations:** ^1^Department of Mathematics, University of Iowa, Iowa City, Iowa 52242,; ^2^Department of Biology, University of Washington, Seattle, Washington 98195,; ^3^Department of Neurosurgery, and; ^4^Iowa Neuroscience Institute, University of Iowa, Iowa City, Iowa 52242

**Keywords:** auditory streaming, bistable perception, spatial cortical maps, spatiotemporal patterns

## Abstract

A key challenge in neuroscience is understanding how sensory stimuli give rise to perception, especially when the process is supported by neural activity from an extended network of brain areas. Perception is inherently subjective, so interrogating its neural signatures requires, ideally, a combination of three factors: (1) behavioral tasks that separate stimulus-driven activity from perception per se; (2) human subjects who self-report their percepts while performing those tasks; and (3) concurrent neural recordings acquired at high spatial and temporal resolution. In this study, we analyzed human electrocorticographic recordings obtained during an auditory task which supported mutually exclusive perceptual interpretations. Eight neurosurgical patients (5 male; 3 female) listened to sequences of repeated triplets where tones were separated in frequency by several semitones. Subjects reported spontaneous alternations between two auditory perceptual states, 1-stream and 2-stream, by pressing a button. We compared averaged auditory evoked potentials (AEPs) associated with 1-stream and 2-stream percepts and identified significant differences between them in primary and nonprimary auditory cortex, surrounding auditory-related temporoparietal cortex, and frontal areas. We developed classifiers to identify spatial maps of percept-related differences in the AEP, corroborating findings from statistical analysis. We used one-dimensional embedding spaces to perform the group-level analysis. Our data illustrate exemplar high temporal resolution AEP waveforms in auditory core region; explain inconsistencies in perceptual effects within auditory cortex, reported across noninvasive studies of streaming of triplets; show percept-related changes in frontoparietal areas previously highlighted by studies that focused on perceptual transitions; and demonstrate that auditory cortex encodes maintenance of percepts and switches between them.

**SIGNIFICANCE STATEMENT** The human brain has the remarkable ability to discern complex and ambiguous stimuli from the external world by parsing mixed inputs into interpretable segments. However, one's perception can deviate from objective reality. But how do perceptual discrepancies occur? What are their anatomical substrates? To address these questions, we performed intracranial recordings in neurosurgical patients as they reported their perception of sounds associated with two mutually exclusive interpretations. We identified signatures of subjective percepts as distinct from sound-driven brain activity in core and non-core auditory cortex and frontoparietal cortex. These findings were compared with previous studies of auditory bistable perception and suggested that perceptual transitions and maintenance of perceptual states were supported by common neural substrates.

## Introduction

**Figure 1.**
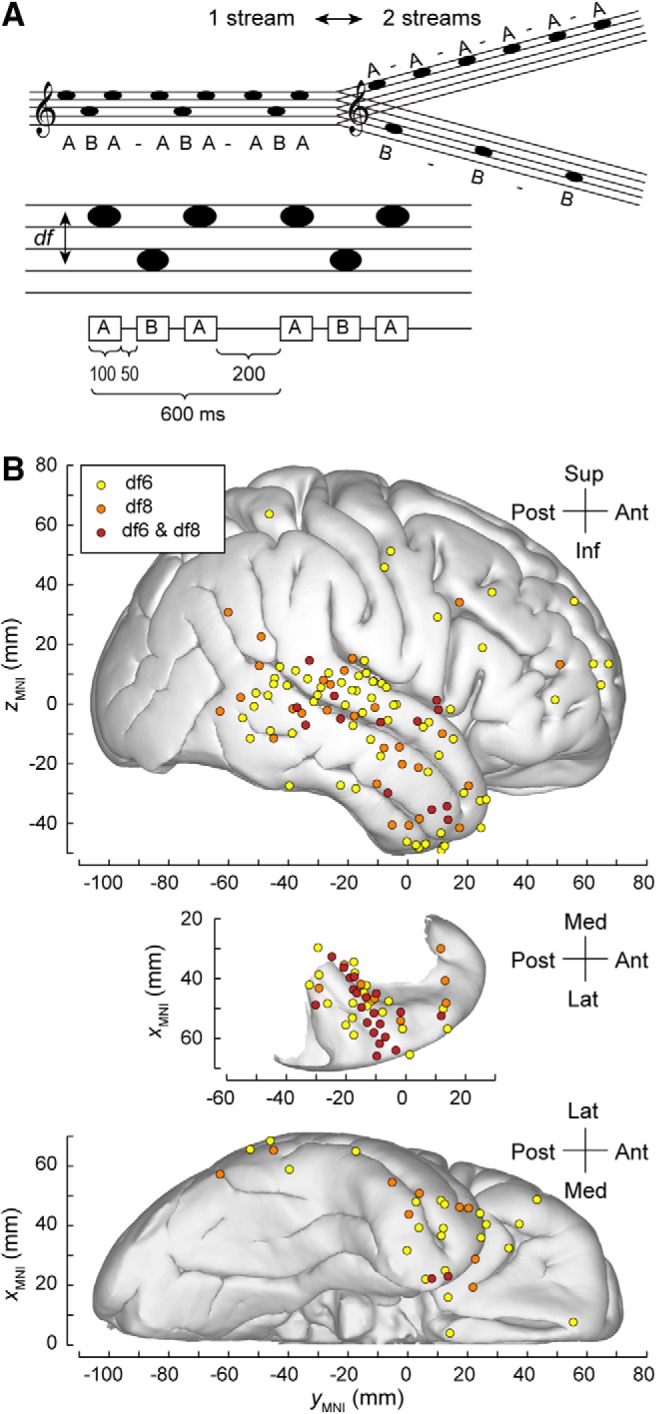
Auditory streaming of triplets and bistable perception. ***A***, Stimuli are sequences of triplets *ABA*_ of pure tones *A* and *B* separated in frequency by df semitones. In humans, switches between percepts 1-stream and 2-stream occur on the order of seconds to tens of seconds. ***B***, Percept-related differences in the AEP as revealed by large-scale human intracranial recordings for bistable stimuli. Topography of significant AEP differences between 1-stream and 2-stream percept at 6 semitones difference, 8 semitones difference, or both stimulus conditions (plotted in yellow, orange, and maroon, respectively). Summary of data from 8 subjects, plotted in MNI coordinate space and projected onto Freesurfer average template brain. Projection is shown on the lateral, top-down (superior temporal plane), and ventral views (top, middle, and bottom, respectively). Sites in the temporal pole and sites in inferior temporal gyrus are shown in both the lateral and the ventral view.

Multistable perception is a class of phenomena in which a single physical stimulus admits two or more mutually exclusive perceptual interpretations. Visual illusions inspired decades-long research on perceptual bistability (Levelt, 1968; Leopold and Logothetis, 1999; and many others), but multistable percepts were also demonstrated in other sensory modalities, including touch (Carter et al., 2008) and audition (van Noorden, 1975). In particular, one task, known as the auditory streaming task, was shown to produce spontaneous switching between two auditory percepts (van Noorden, 1975; Pressnitzer and Hupé, 2006). The stimulus comprises sequences of streaming triplets *ABA*_, where tones of different frequencies *A* and *B* are presented in repeating patterns. Listeners hearing the sequence of triplets perceive either a single coherent auditory stream (1-stream) or two simultaneous distinct streams (2-stream), as shown schematically in Figure 1*A*. The temporal dynamics of these perceptual alternations are similar to those observed for bistable visual stimuli. Their dominance durations follow gamma-like distributions (Pressnitzer and Hupé, 2006) and yield comparable measures of higher statistics, such as skewness, coefficient of variation, and scaling properties (Cao et al., 2016). Moreover, theories of bistable auditory and visual perception share common principles, such as competition, adaptation, and predictive-coding or evidence accumulation (Micheyl et al., 2005; Denham and Winkler, 2006; Winkler et al., 2012; Barniv and Nelken, 2015; Rankin et al., 2015). If functionally similar neural mechanisms underlie perceptual organization across different sensory modalities, the question arises as to what are the anatomical substrates of multistable perception.

The literature on nonvisual perceptual transitions in humans has identified several sensory and frontoparietal areas, including bilateral activation of auditory cortex (AC), posterior insular cortex, supramarginal gyrus (Kondo and Kashino, 2009), intraparietal sulcus (Cusack, 2005), inferior frontal gyrus (IFG), and anterior cingulate cortex (Kondo and Kashino, 2007; Basirat et al., 2008). On the other hand, evoked responses during the perceptual states induced by streaming of triplets, and their corresponding differences, have been localized to AC by MEG and EEG recordings (Gutschalk et al., 2005; Snyder et al., 2006; Hill et al., 2012; Billig et al., 2018; Sanders et al., 2018), and to AC and intraparietal sulcus by fMRI research (Cusack, 2005; Hill et al., 2011).

In this study, we used recordings from the human brain to characterize features of neural dynamics associated with bistable percepts. We focused on auditory perception and identified neural substrates that discriminate between perceptual states during alternation cycles. Electrocorticography (ECoG) data were collected from 8 neurosurgical patients while they listened to sequences of repeated triplets and reported their perception (Fig. 1*A*). Spatiotemporal activation patterns showing differential responses to the mutually exclusive percepts were identified across several cortical areas (Fig. 1*B*; summary of group data). Significant differences in the averaged auditory evoked potential (AEP) calculated for 1-stream and 2-stream percepts were found at recording sites within core and non-core AC, surrounding auditory-related temporoparietal cortex, and frontal areas. Many of these areas overlap with ROIs from previous reports that showed activation time-locked to the perceptual switches. A group-level analysis was performed to test whether the same neural substrates may support the maintenance of the bistable percepts and the transitions between them. ECoG recordings were projected on a one-dimensional embedding space, and the analysis was run on this feature rather than on the spatially distributed time-series. Activation of the AC was found to capture differences between 1-stream and 2-stream percepts during their sustained states as well as at times immediately before the perceptual changes.

## Materials and Methods

### 

#### Participants

Eight neurosurgical patients treated for pharmaco-resistant epilepsy participated in the experimental sessions (5 males and 3 females; age range 21–47 years; median 32 years; herein identified as B335, L357, R369, L372, R376, R399, L409, and R413). All subjects satisfied the following selection criteria for inclusion in the study: (1) extensive electrode coverage of ROIs within temporal lobe, including core and non-core AC, and additional electrode coverage of frontal and parietal lobes; (2) good behavioral performance (>85% accuracy) to the control stimuli (see below) and (3) adequate behavioral response during perceptually ambiguous conditions (number of perceptual switches within typical range based on published reports for healthy populations, and sufficiently many to ensure meaningful statistical analysis); (4) epileptic zones outside ROIs (sites implicated in seizure activity were excluded); and (5) normal hearing and no significant cognitive deficits as determined by standard audiological and neuropsychological testing. An additional control group of 21 healthy subjects (10 males, 11 females; ages 19–45 years; median 25 years) was recruited to obtain behavioral data in the same task. Variability of responses between individuals in the control group of healthy subjects was characterized as mean, median, and range of percept mean-durations per condition as well as range for the number of switches and durations during individual experimental blocks. Behavioral data of all ECoG subjects whose recordings were included in the analysis fell within the range of individual block-based percept durations of the healthy control group.

#### Stimuli

Stimuli were 5-min-long sequences of pure tones, presented in a triplet repetition paradigm *ABA*_*ABA*_… with tones *A* and *B* separated in frequency by df semitones and the underscore denoting a silent gap. Tones were 100 ms in duration, gated with 10 ms raised cosine ramps, and separated by 50 ms silent intervals within triplet and 200 ms of silence between triplets. The stimuli were thus characterized by a 300 ms stimulus onset asynchrony between successive A tones and 600 ms between successive B tones. Each triplet was 600 ms in duration, and each experimental block consisted of 500 triplet repeats (Fig. 1*A*). In all subjects except R369, tone *B* had a frequency *f_B_* = 1000 Hz whereas tone *A* had a frequency *f_A_* = 1122, 1414, 1587, and 2000 Hz corresponding to df = 2, 6, 8, or 12 semitone differences, respectively. In Subject R369, stimulus frequencies were *f_B_* = 1250 Hz and *f_A_* = 1403, 1768, 1984, and 2500 Hz for 2, 6, 8, or 12 semitones above *B*, respectively.

#### Recordings

ECoG recordings were obtained simultaneously from multicontact depth electrodes and subdural electrode arrays. All electrodes were placed solely on the basis of clinical requirements to identify seizure foci (Reddy et al., 2010; Nagahama et al., 2017). Electrode arrays were manufactured by Ad-Tech Medical and PMT. Electrode implantation, recording and ECoG data preprocessing have been previously described in detail (e.g., Howard et al., 1996, 2000; Reddy et al., 2010; Nourski and Howard, 2015). In brief, depth electrode arrays (8–12 macro contacts, spaced 5 mm apart) targeting Heschl's gyrus (HG) were stereotactically implanted along the anterolateral-to-posteromedial axis of the gyrus. Additional arrays targeted insular cortex and provided coverage of posteromedial HG (HGPM), anterolateral HG (HGAL), planum temporale (PT), planum polare (PP), insula, and superior temporal sulcus (STS). Subdural grid arrays were implanted over the lateral hemispheric surface, including superior temporal gyrus (STG), middle temporal gyrus (MTG), supramarginal gyrus (SMG), postcentral gyrus (PoCG), precentral gyrus (PreCG), middle frontal gyrus (MFG), and IFG. Subdural grid arrays consisted of platinum-iridium disc electrode contacts (2.3 mm exposed diameter, 5–10 mm interelectrode distance) embedded in a silicon membrane. In all subjects, a subgaleal electrode was used as a reference. ECoG data acquisition was performed using a RZ2 real-time processor (Tucker-Davis Technologies) in Subjects B335 and L357 and a Neuralynx Atlas System in all other subjects. Collected ECoG data were amplified, filtered (0.7–800 Hz bandpass, 12 dB/octave rolloff), digitized at a sampling rate of 2034.5 Hz (Tucker-Davis Technologies) and 2000 Hz (Neuralynx), and stored along with timing of button-press events for subsequent offline analysis. Locations of recording sites were confirmed by coregistration of preimplantation and postimplantation structural imaging and aided by intraoperative photographs (Nourski and Howard, 2015). Preimplantation whole-brain high-resolution MRI scans (T1-weighted structural MRIs, resolution 0.78 × 0.78 mm, slice thickness 1.0 mm) and postimplantation thin-sliced volumetric CT scans (resolution 0.51 × 0.51 mm, slice thickness 1.0 mm) were coregistered using a linear algorithm with 6 degrees of freedom (Jenkinson et al., 2002). Before analysis, all recording sites implicated in seizure activity (in the epileptic zone; see Table 1) were excluded. Herein, only the remaining contacts were called recording sites or recording contacts.

**Table 1.** ECoG group patient data*^a^*

Subject	Age (years)	Gender	Dominant hand	Seizure focus	Preoperative medication	Medication within 24 h before recording
B335	33	M	R	Bilateral	Lamicital 500 mg	Levetiracetam
				medial	Keppra XR 2500 mg	1000 mg
				temporal lobe		
L357	35	M	R	Left posterior	Lacosamide 2 × 200 mg	No medication
				hippocampus	Levetiracetam 2 × 1000 mg	administered
R369	29	M	R	Right medial	Lorazepam 2 × 0.5 mg	No medication
				temporal lobe	Levetiracetam 2 × 200 mg	administered
					Topiramate 2 × 250 mg	
L372	33	M	R	Left temporal	Lorazepam 2 mg (after seizure)	Levetiracetam
				pole	Levetiracetam 1500 mg/d	2 × 1500 mg
					Topiramate 200 mg/d	Topiramate
						2 × 100 mg
R376	47	F	R	Right medial	Divalporex 1000 mg (as needed)	Gabapentin
				temporal lobe	Gabapentin 3 × 600 mg	2 × 600 mg
					(after week 9)	
					Zonisamide 5 × 100 mg	
R399	21	F	R	Right temporal	Lamotrigine 600 mg/d	No medication
				lobe with early	Levetiracetam 2000 mg/d	administered
				propagation to	Lorazepam 2 mg (as needed)	
				right inferior		
				lateral frontal		
				lobe		
L409	31	F	L	Left medial	Lacosamide 200 mg/d	No medication
				temporal lobe	Levetiracetam 1500 mg/d	administered
					Clonazepam 2 mg (as needed)	
R413	21	M	L	Right medial	Oxcarbazepine 1050 mg/d	Oxcarbazepine
				temporal lobe	Levetiracetam 2000 mg/d	3 × 600 mg

*^a^*Research data were not sampled from sites implicated in seizure activity.

#### Experimental design and statistical analyses

##### Experimental protocol.

The experimental tasks supported perceptual bistability in auditory streaming (Cusack, 2005; Gutschalk et al., 2005; Pressnitzer and Hupé, 2006). Participants underwent either one or two experimental sessions of three blocks each: df2-12, df6, and df8. During block df2-12, sequences of triplets of tones at df = 2 were interleaved with sequences of triplets of tones at df = 12. This 5 min stimulus was used as a control condition to elicit stable 1-stream (*ABA*_*ABA*_ …) and 2-stream (*A*_*A*_*A*_*A*_ … and _*B*_*B*_…) percepts, respectively. In total, block df2-12 consisted of 24 percept durations spanning 5–38 and 9–45 triplets per df2 and df12, respectively, with means of 11.9 s and 13.1 s, and no significant difference in medians (*p* = 0.8985, two-sided Wilcoxon rank-sum test). Perceptually bistable stimuli were obtained by presenting 500 repeats of triplet *ABA*_ with 6-semitone separation between tones *A* and *B* (condition df6) or 500-triplet repeats based on 8-semitone difference (condition df8). Acoustic stimuli were delivered through earphones integrated into custom-fitted ear molds similar to those worn by hearing aid users. Subjects were instructed to report the emergence of 1-stream and 2-stream alternating percepts (i.e., perceptual changes) by pressing a button on a response box. Experiments were performed during chronic invasive monitoring, in a dedicated electrically shielded suite in the University of Iowa Clinical Research Unit. Research protocols were approved by the University of Iowa Institutional Review Board and the National Institutes of Health, and written informed consent was obtained from all subjects. Research participation did not interfere with acquisition of clinically necessary data, and subjects could rescind consent for research without interrupting their clinical management.

##### Behavioral data analysis.

All percept durations during bistable stimuli, except for the first in each block, were included in behavioral analysis. The mean dominance durations for each percept type were computed per block, subject, and condition separately. Individual percept durations were normalized to these values, and histograms were constructed for 1-stream and 2-stream percepts in df6 and df8 conditions, for the healthy and the ECoG subject group. Mean values per ECoG blocks were compared with means computed for healthy subjects.

##### ECoG data analysis.

All ECoG subjects, except Subject R413, had three blocks from the same experimental session included in the analysis of neural data (blocks df6 and df8 of bistable stimuli together with block df2-12 of the control stimulus). Subject R413 reported no switch in perception during block df8. For that subject, only blocks df6 and df2-12 were selected for further analysis.

##### Partition of data in triplet-locked epochs.

Before analysis, ECoG data from each recording site were downsampled to 1000 Hz and then denoised using an adaptive filtering procedure based on the demodulated band transform approach (Kovach and Gander, 2016). The signal underwent an automated screening process for possible contamination from electrical interference, epileptiform spikes, high-amplitude and slow-wave activity, and movement artifacts. For local field potential (LFP) analysis, the data were bandpass filtered between 1.5 and 70 Hz. Frequency components near 2.5 Hz (from 2.2 to 2.7 Hz) were also eliminated by temporal and spatial filters. In particular, the spatial filter was constructed by discarding the first 30 components in the singular value decomposition of the normalized spatial correlation matrix defined over all recording sites, in the narrow frequency band above. The filtering step was used to stabilize the baseline of the ECoG signal for subjects that had their data collected with the Neuralynx system; for consistency, it was implemented across the entire ECoG group. A rejection criterion was then applied to each data acquisition channel, for any given triplet. The rejection criterion was defined by voltage exceeding 4 SDs of the within-block mean. For each 5-min-long stimulus presentation and each recording site, the ECoG signal was divided into 600 ms triplet-locked epochs (hereafter to be called “trials”), of 500 total. Time *t* = 0 of every such trial corresponded to the onset time of the individual *ABA*_ triplet. The subject's response indicating the percept was used to label each trial accordingly: following the approach of Gutschalk et al. (2005), trials during dominance durations of percepts reported as 1-stream and 2-stream were placed in perceptual categories 1 and 2, respectively. Trials identified by the above rejection criteria and those preceding the first reported perceptual switch during bistable condition were excluded from analysis. A number of epochs immediately preceding each button press were also discarded (2 for B335, L357, R413; 3 for L372, R376, L409; 4 for R399; 6 for R369) to account for the subject's average reaction time (RT) calculated from the latency of their behavioral response to control stimuli (RTs for those subjects were ∼0.6, 1.34, 2, and 3.22 s, respectively). The statistical analysis of ECoG data was trial-based and was performed using custom software in MATLAB (The MathWorks).

##### Percept-related differences in the AEP.

Trials were split in sets *S*_1_ and *S*_2_ according to the subject's reported percept, as described above, and time τ spanned the length of a triplet (0–600 ms). Then the averaged evoked potentials AEP_1_(*j*;τ) and AEP_2_(*j*;τ) were computed at each recording site *j* separately over *S*_1_ and *S*_2_. A two-sample, two-tailed *t* test was performed to identify differences between AEP_1_ and AEP_2_ at each time point and recording site. To reduce the number of comparisons and control the family-wise error rate, a cluster-based permutation nonparametric test and a false discovery rate (FDR) algorithm were also implemented.

##### Permutation test based on the maximum cluster-level mass.

The algorithmic steps proposed by Maris and Oostenveld (2007) were implemented on a site-by-site basis. The relationships between different recording sites were no longer preserved under the permutation process. The clusters were constructed only based on adjacent time points. Maximum cluster-level mass histograms were constructed for each site separately based on *n* = 10,000 permutations of the corresponding ECoG data. The permutations were generated by randomly assigning percept labels 1 and 2 to trials. For each such random partition (Ŝ_1_,Ŝ_2_), the *t* values *t_j_*_,τ_ were computed at every recording site and time point. All *t_j_*_,τ_ corresponding to an uncorrected *p* value <0.05 were kept, whereas the others were ignored. Then remaining *t* scores were grouped into clusters based on adjacent time points, separately for each contact. Only clusters with a sufficient number of adjacent *t* scores (at least 20) were retained, and the rest were discarded. In other words, only clusters spanning time windows at least 20-ms-long were included in the analysis. Next, the cluster-level mass *t* score was computed as the sum of *t_j_*_,τ_ over all time points τ belonging to the cluster. Then, at any given permutation, the *t_cls_* statistic was defined as the most extreme cluster-level mass *t* score (maximum in absolute value). The histogram of all 10,000 permutations-based *t_cls_* values was constructed. In the end, the cluster-level masses *t_c_*_;_*_obs_* of the observed data were computed following the same procedure, and they were compared with the null hypothesis distribution, separately for each recording site. The Monte Carlo *p* value estimate *p_c_*_;_*_obs_* = (*r* + 1)/(*n* + 1) was derived from the proportion *r*/*n* of the *t_cls_* distribution that exceeded the observed statistic *t_c_*_;_*_obs_* (North et al., 2002, 2003; Harrison, 2012). Then the *p* value of the entire cluster was assigned to each of its members resulting in adjusted *p* values *p_j_*_,τ_. For all points outside of these clusters, the adjusted *p* value was set to 1. Percept-related differences in AEP_1_(*j*;τ) and AEP_2_(*j*;τ) were considered significant if *p_j_*_,τ_ was smaller than or equal to the critical α level 0.05.

##### FDR correction for multiple comparisons.

The cluster-based permutation test was run for all recording sites, tone frequency separation conditions (df2-12, df6, df8 for all except R413, and df2-12, df6 for R413), and all 8 ECoG subjects. Several clusters could be found per recording site. The *p* value at each site per experimental block (*p_j_*) was taken as the minimum across the whole triplet time window of the *p* values *p_j_*_,τ_ from the cluster-based permutation test. FDR control (Benjamini and Krieger, 2006) was applied on these recording-site *p* values across all sites and each set of the control and bistable conditions, per individual subject. Up to 1% of false positives were allowed that corresponded to an average of ∼8 contacts potentially wrongly selected by the statistical analysis. Only clusters that remained significant following the FDR correction were reported.

##### Percept classification with support vector machine (SVM).

Feature selection and SVM algorithms were used to examine how well spatially distributed LFP signals had captured differences between 1-stream and 2-stream at individual trials (triplets) for each subject. ECoG data were preprocessed as described for the univariate analysis. Then trials were split in two classes according to perception, and the feature space was defined as the collection of 12 50-ms-wide nonoverlapping time windows spanning the triplet time at all recording sites per subject and experimental block. Average LFP values over each time bin and site were computed and used as input to the classifier. Four-fifths of the data were randomly selected as the “training” set, and the remaining data were assigned to the “test” set. The data at each feature were scaled to its *z* score over the test and training set, separately. For some of the experimental blocks included in the classification, trials associated with 1-stream and 2-stream were not in 1:1 ratio. Instead, they were in approximate ratio of 1:3 (for L372), 2:1 (R399), 1:2 (R413) at df6, and 1:3 (L357, R376), 1:2 (L372, L409), 2:1 (R399) at df8, respectively. We applied the method introduce by Wei and Dunbrack (2013) to compensate for such disparity and to obtain an unbiased training set. A feature selection iterative procedure was also implemented in the training stage of the classifier. This was done to control for data redundancy due to the high dimensionality of the feature space (12× number of recording sites) analyzed over a much smaller size data sample (number of trials < 500). The *F* score of every feature was calculated using the training set (Chen and Lin, 2006). The features were ordered from the largest to the smallest *F* score, and only the top *D* of them were kept (*D* was chosen at 10% of the size of the training set). SVM was applied recursively to training data as follows: the algorithm was repeated for *k* = *D*, *D* − 1, …, 1, so that, at each step *k*, one feature was dropped from the analysis (the feature with the lowest *F* score); the training set was split into a subtraining set and a validation set; then SVM was used to train the subtraining set to obtain a predictor and predict the valid set. This internal loop was repeated 5 times, and the average validation error was calculated. Finally, the optimal feature-dimension D* was defined as the smallest of all *k* at which the lowest average validation error was determined. Once the optimal features were identified over the training set, SVM was applied to the test set and the accuracy of the classification was computed. If the test set was unbalanced, then the test accuracy was adjusted according to the balanced test accuracy formula proposed by Wei and Dunbrack (2013). Multiple repetitions (100 times) of the paired feature selection-SVM algorithm (i.e., the training testing steps) were used to generate a distribution of accuracies. For each particular feature (i.e., 50 ms bin at a given recording site), the probability of it belonging to an optimal feature set was computed. Features with probability >0.3 were considered meaningful to the estimation of the test accuracy and therefore retained. The sites identified by the procedure were then used to generate the classifier-driven spatial map of LFP differences between trials in 1-stream and 2-stream for the control and bistable stimuli.

##### Spatial maps across subjects.

MNI coordinates of all recording sites identified by the univariate analysis in either the control or the two bistable conditions, and across all 8 ECoG subjects were used to construct the group spatial map. The same approach was used to summarize the spatial distribution of all sites identified as most probable optimal features by the classification analysis, across all 8 ECoG subjects. These were obtained by projection of all sites onto the right hemisphere of the average template brain using the structural MRI analysis software FreeSurfer (Athinoula A. Martinos Center for Biomedical Imaging, Charlestown, MA). Left hemisphere contacts were projected onto the right hemisphere by multiplying their MNI *x* coordinate by −1.

##### High gamma event-related band power (ERBP).

Quantitative analysis of the ERBP was also performed with the focus on the high gamma frequency band (70–150 Hz). High gamma activity has been reported in several studies to be a robust indicator of local cortical function (Nourski et al., 2013, 2014a,b). ECoG data were downsampled to 1000 Hz, denoised, screened for artifacts, and bandpass filtered between 70 and 150 Hz. The power envelope signal was computed by Hilbert transform, log-transformed, normalized to the mean log-power over the entire 5-min-long neural signal taken as reference, and smoothed by bandpass filter over 1.5–40 Hz range. The continuous ERBP signal was then split into 600-ms long trials, which were labeled as 1-stream and 2-stream perceptual classes based on behavioral reports. A rejection criterion for trials, defined by ERBP signal exceeding 4 SDs of the within-block mean, was also applied to each acquisition channel. The ERBP data were then analyzed statistically by the cluster-level mass permutation test with FDR correction as described for LFP data above.

##### Group-level statistics using low-dimensional embeddings.

Two hypotheses were examined. First, we tested that LFPs recorded from the AC uncovered differences between 1-stream and 2-stream at individual trials during the maintenance of bistable percepts. Here we used the same approach as in the univariate analysis and discarded a number of epochs immediately preceding each button press. Then we tested that LFPs obtained from the same recording sites did likewise reveal 1-stream and 2-stream differences at trials before the switch between percepts. The comparison included one trial per button press; the trial was chosen to correspond to the last complete triplet heard by the subject before they reported the perceptual change; the trials from all subjects were combined to ensure sufficient statistical test power. The analysis was performed on all recordings obtained from the contacts placed in core (HGPM) and non-core (HGAL, PT, PP, STG) AC. It included data from all 8 ECoG subjects and was done for each block df2-12, df6, and df8 separately. The number of contacts per subject were as follows: 35 (B335), 17 (L357), 44 (R369), 44 (L372), 38 (R376), 34 (R399), 12 (L409), and 51 (R413) (Table 2). Data from non-core auditory areas within the superior temporal plane (HGAL, PT, PP) were combined for the purpose of this analysis. To reduce noise and to ameliorate the impact of intercortical and interindividual variability across the group, single-trial LFPs were standardized and projected onto a one-dimensional embedding space using diffusion maps and manifold learning techniques (Coifman and Lafon, 2006; Nadler et al., 2006; Pfau et al., 2013; Mishne et al., 2016). No knowledge about which perceptual categories the trials belonged to was assumed at this stage. Finally, once all trials were projected on the embedding space, they were labeled according to the subject's report. Wilcoxon rank-sum tests were performed on the embedded projections to determine whether the two percept-related subsets were drawn from the same distribution. Rejection of the null hypothesis was taken as indication of statistically significant differences between 1-stream and 2-stream trials.

**Table 2.** Number of ECoG recording sites showing AEP differences at statistical significance between 1-stream and 2-stream per subject and condition, and anatomical lobe (*m* contacts out of *n* total per recording area; *m*/*n*)*^a^*

	B335	L357	R369	L372	R376	R399	L409	R413	Total
No. of sites	*66*/157	*28*/148	*63*/225	*64*/191	*57*/210	*24*/195	*9*/170	*65*/223	*376*/1519
	**36**/157	**1**/148	**31**/225	**31**/191	**1**/210	**15**/195	**1**/170	21/223	**137**/1519
	**38** /157	**2**/148	**10** /225	**7**/191	**3** /210	**16**/195	**2**/170		**78**/1296
Temporal									
HGPM	*6*, **6,5**/6	*5*, **1**,−/5	*8*, **4,2**/8	*6*, **4,1**/6	*7*, −,−/7	*3*, −,−/3	*1*, −,−/1	*7*, **1**/8	*43*, 1**6, 8**/44
HGAL	*10*, **10,8**/10	*3*, −,**2**/5	*5*, **3,1**/5	*4*, **3**,−/4	*4*, −,−/4	−, −,−/4		*4*, **1**/4	*3*0, **17, 11**/36
PT	*3*, **3,3**/3		*4*, −,−/4	*4*, **1**,−/4	*3*, −,−/3	*2*, −,−/2		*4*, −/5	*20*, **4, 3**/21
PP	*3*, **1,2**/5		*2*, **1**,−/6	*2*, **1**,−/4	*3*, −,−/3	−, −,**1**/1		*1*, −/3	*11*, **3, 3**/22
STG	*7*, **5,5**/11	*3*, −,−/7	*16*, **13,3**/21	*15*, **9,2**/26	*15*, −,−/21	*5*, **2,1**/24	*2*, −,**1**/11	*15*, **5**/31	*78*, **34, 12**/152
MTG	*6*, **3,3**/15	*4*, −,−/17	*8*, −,**1**/36	*5*, **1,1**/21	*3*, −,**1**/42	*4*, **3,5**/36	−, −,**1**/13	*10*, **3**/55	*40*, **10, 12**/235
ITG	*2*, −, **1**/10	*1*, −,−/8	*1*, −,−/8	−, −,**1**/7	*1*, −,−/14	*2*, **2,2**/13	−, −,−/7	*7*, **2**/17	14, **4, 4**/84
STS	*3*, **2,2**/10	*3*, −,−/3	*1*, **1**,−/3	*1*, **2**,−/5	*2*, −,−/9	−, **1,1**/4	*1*, −,−/5	*1*, −/2	*12*, **6, 3** /41
INS	*5*, −,**3**/10	−, −,−/5	*1*, −,−/1	*2*, −,−/2	*2*, −,−/4		*2*, −,−/2	−, −/3	*12*, −, **3**/27
TP	*5*, **2,1**/20	−, −,−/11	*2*, **6,1**/13	−, **1**,−/3	−, −,−/7	−, **1,3**/15	−, −,−/11	*1*, **3**/10	*8*, **13, 5** /90
PHG	*1*, −, −/3	−, −,−/2	−, −,−/3	*1*, **1**,−/5	−, −,−/2	−, −,−/5	−, −,−/1	*1*, −/3	*3*, **1**, −/24
FFG	*1*, −,−/3	−, −,−/7	−, −,−/4	*2*, −,−/4	−, −,−/2	−, −,−/5	−, −,−/4	*1*, −/3	*4*, −, −/32
Parietal									
SMG	*1*, **1,1**/5	*1*, −,−/4	*4*, −,−/17	*5*, −,**1**/22	*5*, −,−/13	*3*, −,−/9	−, −,−/6	*2*, **1**/6	*21*, **2, 2**/82
ANG	−, −,−/2	*1*, −,−/6	*2*, −,**1**/24	*1*, **1**,−/10	*1*, −,1/9		−, −,−/12	−, −/9	*5*, **1, 2**/72
PoCG	*1*, −,**1**/2	−, −,−/3	*1*, −,−/2	*3*, **3**,−/9	*4*, −,−/6	−, −,−/3	*1*, **1**,−/12		*10*, **4, 1** /37
Frontal									
PreCG		*4*, −,−/9	*3*, **1**,−/11	*6*, **2**,−/9	*1*, −,−/6	*1*, −,−/7	−, −,−/10	*2*, −/6	*17*, **3**, −/58
TFP		−, −,−/4	−, −,−/1	−, −,−/3	*1*, −,−/5	−, **1**,−/2	−, −,−/4	*3*, **2**/8	*4*, **3**, −/27
MFG		−, −,−/14	−, **1,1**/15	−, −,−/15	*1*, −,**1**/17	−, **1**,−/18	−, −,−/7	*1*, **1**/18	*2*, **3, 2**/104
IFG	−, −,−/1	*2*, −,−/6	−, −,−/12	−, **1**,−/11	*1*, **1**,−/6	*2*, **2,1**/9	−, −,−/7	−, −/8	*5*, **4, 1**/60
GREC	−, **1**,−/7	*1*, −,−/5	*1*, −,−/4	*1*, −,−/2	−, −,−/1	−, −,−/3	−, −,−/3	*2*, −/6	*5*, **1**, −/31
OFC	*6*, **1**,−/16	−, −,−/10	*3*, **1**,−/19	−, −,−/8	*1*, −,−/10	*2*, **1,2**/17	−, −,−/16	*1*, 2/7	*13*, 5, **2** /103
Other sites									
AMYG	−, −,**3**/8	−, −,−/2	−, −,−/4	*2*, **1**,−/2	*1*, −,−/3	−, **1**,−/7			*3*, **2, 3**/26
HIP	*6*, **1**,−/10	−, −,−/2		*1*, −,**1**/6	−, −,−/2	−, −,−/2			*7*, **1, 1**/22
Other		−, −,−/13	*1*, −,−/4	*3*, −,−/ 3	*1*, −,−/21	−, −,−/6	*2*, −,−/38	*2*, −/ 11	*9*, −, −/97

*^a^*Differences in percept-related AEP at the control (bistable) stimulus were marked in italics (bold) font. Subject prefix B/L/R indicates the side of electrode coverage (bilateral/left/right hemisphere). Sites with statistically significant AEP differences in control (in italics) versus bistable (in bold; df6 and df8) conditions. INS, Insula; PHG, parahippocampal gyrus; FFG, fusiform gyrus; ANG, angular gyrus; TFP, transverse frontopolar gyrus; GREC, gyrus rectus; AMYG, amygdala; HIP, hippocampus.

Group-level statistics were performed using the following diffusion maps. First, ECoG data were preprocessed as described for the univariate analysis with one exception: for each 5-min-long stimulus presentation and each recording site, 500 LFP trials were obtained by extracting 2-s-long time windows around each triplet-locked epoch. In other words, for the group-level analysis, each trial consisted of a larger time window that covered not only the triplet-locked epoch but also its nearby triplets in the *ABA*_*ABA*_ sequence; the time range consisted of 700 ms pretriplet, 600 ms triplet, and 700 ms post-triplet epoch times. Each trial was assumed to inherit the perceptual category label 1-stream or 2-stream from the triplet-locked epoch at its center. The 2 s time window was chosen arbitrarily; other trial durations were also tested, and they were found to generate equivalent conclusions (data not shown). Second, at each recording site *j* and for each trial *t*, the LFP sample *X*(*t*; *j*; τ) was normalized. The mean and SD of *X*(*t*; *j*; τ) were computed with respect to time τ spanning the length of the trial (0–2000 ms); then the *z* score was determined, *z*(*t*; *j*; τ) = (*X*(*t*; *j*; τ) − μ)/σ. Third, for each cortical area separately, cosine similarities cos(θ*_tt̃_*) with angle restricted between 0 and 90 degrees were calculated for each pair of *z* scores of all 500 trials. Fourth, the cosine similarity matrix was transformed into the affinity kernel (*a_tt̃_*) by means of the nonlinear map *a_tt̃_* = exp(− λ tan θ*_tt̃_*), where λ is a scaling factor. The exponential kernel enhanced locality in the space of trials as it assigned a maximum affinity value of 1 to collinear samples, a negligible affinity (close to zero) to orthogonal samples, and subunitary values for everything else in between. A scaling factor of λ = 0.2 (B335, L357, R369, R376, R399) and λ = 0.1 (L372, L409, R413) brought the embeddings of the entire ECoG group and of all blocks df2-12, df6, df8 to a comparable numerical range from −0.6 to 0.6. Finally, a unique affinity matrix *a* was computed per subject and experimental block. It was defined as the mean of the affinity kernels *a_HGPM_*, *a_HGAL_*_,_*_PT_*_,_*_PP_*, and *a_STG_*. The first nontrivial eigenvector of matrix *a* was used to construct the one-dimensional embedding of trials. Then data of all embeddings were analyzed by the Wilcoxon rank-sum statistics at 0.05 significance level per df2-12, df6, and df8 conditions separately. The *p* values were determined for each subject as well as for the aggregated group data, and corrected at 1% FDR.

## Results

### Behavioral task performance

**Figure 2.**
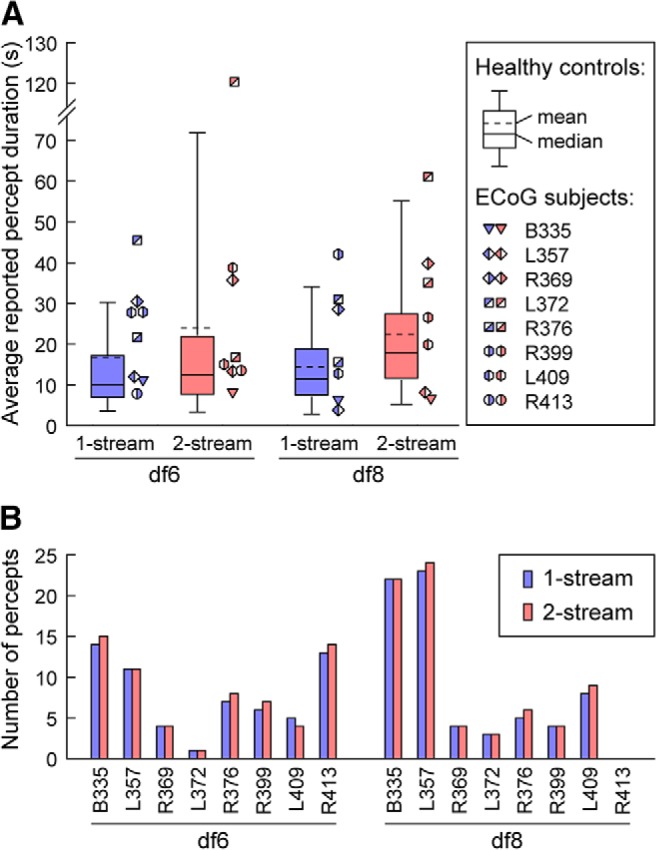
***A***, Average percept durations (in seconds) for 1-stream (blue) and 2-stream (red) and bistable stimuli df6 and df8 shown for the control healthy population group (boxplots) and the ECoG group (symbols). The plots include the fifth, 25th, 50th (median), 75th, and 95th percentiles as well as the mean (dashed line) over the average values from the control group. For the ECoG subjects, the mean percept duration is shown only for the experimental blocks used in the ECoG data analysis (one block per subject and df condition). ***B***, Number of percepts (excluding the first) per type and condition, in each experimental block used in the ECoG data analysis. Subject R413 reported at df8 a 2-stream percept but no subsequent switches.

Behavioral responses to the auditory stimuli in the 8 ECoG subjects were compared with the behavioral data obtained from a control group of 21 healthy subjects who performed the task under the same experimental conditions (same instructions, stimulus delivery, and response recording protocols). The ECoG group exhibited behavioral performance comparable to the perceptual responses of healthy subjects, when taken on a block-by-block basis. The histograms of normalized percepts had similar statistics. For both healthy and ECoG subject group, they were well fit by gamma distributions of mean 1 and shape parameter close to 2, a result consistent with previous reports (Barniv and Nelken, 2015; Rankin et al., 2015; Cao et al., 2016). Large variations were observed in the average percept durations among subjects within and across both groups. This was not surprising given that perceptual switching in auditory streaming of triplets was previously reported to be individual rather than population specific (Denham et al., 2014). The average percept durations of the ECoG subjects typically fell between the fifth and the 95th percentile of the control group data (Fig. 2*A*), and both groups spent a comparable fraction of time in the 1-stream percept during either block df6 and df8 (0.39 for control and 0.43 for ECoG group, on average).

The group mean durations over the ECoG blocks selected for statistical and classification analysis were compared with the group means of the healthy population. ECoG subjects exhibited percept means larger than those of healthy controls, equivalent to a reduction in switching events. Specifically, ECoG group mean durations were 22.9 and 32.6 s for 1-stream and 2-stream at df6 and 19.9 and 28 s at df8, whereas healthy subjects had mean durations of 16 s, 23.6 s at df6 and 13.8 s, 21.9 s at df8 (Fig. 2*A*). Overall, for both percepts and df conditions, these differences were statistically significant with means of 26 and 18.8 s for ECoG and healthy subjects, respectively (*p* = 0.003; two-sided Wilcoxon rank-sum test). They were in line with recent studies that reported slower alternation rates in subjects with brain disorders (Ngo et al., 2011; Aznar Casanova et al., 2013) and studies that investigated how certain drugs or increases in GABA concentration in the cortex affect perceptual switching (Carter et al., 2007; Kondo and Kochiyama, 2017). Following electrode implantation, the ECoG subjects were tapered off of the anticonvulsant epileptic drugs (Table 1); however, the extent to which their specific preoperative medication impacted perception or the neural basis of perception could not be assessed.

RTs and button press accuracy were calculated for ECoG subjects from their response to the control stimulus df2-12. Both percepts can be heard during either df2 and df12 stimuli, but subjects are typically biased toward 1-stream at df2 and toward 2-stream at df12 (van Noorden, 1975). Indeed, Subjects B335, R369, L372, L409, and R413 reported 24 of 24 of the df2-12-induced percepts, at accuracy 100%. Subjects L357, R376, and R399 either missed one of the df2-12-triggered alternations or, to the contrary, identified an additional switch, at accuracies 96%, 96%, and 88%. Their RTs, computed as average latency of response to the change from df = 2 to df = 12 and back over 5-min-long stimulus presentation, were 0.65, 0.36, 3.22, 1.24, 1.29, 2, 1.48, and 0.78 s for Subjects B335, L357, R369, L372, R376, R399, L409, and R413.

While the number of percepts of each type and bistable condition differed among the ECoG subjects (Fig. 2*B*), the overall number of triplets per reported percept and df were similar across the group: mean ± SD number of triplets of 202 ± 64 (222 ± 42) for 1-stream (2-stream) at df6 and 174 ± 51 (269 ± 57) at df8, respectively. Pairwise comparisons of block mean durations per percept type between df6 and df8 for both healthy and ECoG groups were performed. They did not reveal significant differences in behavioral responses to these bistable stimuli (*p* = 0.3069 and *p* = 0.0973 in a two-sided Wilcoxon rank-sum test, for 1-stream and 2-stream respectively, for healthy subjects; and *p* = 0.8665 and *p* = 0.9551 for ECoG subjects). This result was consistent with observations made by Gutschalk et al. (2005) in an MEG study. They used the same pair of stimuli to generate bistable auditory perception and, likewise, found no significant behavioral differences in the amount of streaming at 6 versus 8 semitones.

### Neural responses to control stimuli

At stimulus df2-12, triplets *ABA*_ were grouped in subsequences of two types based on the frequency difference between tones *A* and *B*, 2 semitones versus 12 semitones. All ECoG subjects identified correctly the transition between these inputs and reported integration (1-stream) during sequences of triplets at df = 2 and segregation (2-stream) at df = 12. The 5-min-long ECoG recordings were split into 600 ms trials and binned into perceptual Classes 1 and 2 based on the behavioral response. Trials from the time event of the stimulus switch to the time event of the behavioral response to the switch were excluded. All remaining trials were then compared using a nonparametric statistical permutation test.

Differences in AEPs between 1-stream and 2-stream percepts, at statistical significance for control stimulus df2-12, were identified in primary and nonprimary AC, surrounding auditory-related temporoparietal cortex, and frontal areas (Table 2). Electrodes implanted in HGPM; HGAL, PT, PP, STG; MTG, STS, SMG, insula; PreCG, PoCG; and inferior temporal gyrus (ITG) showed AEP differences consistently across subjects. Recording sites showing differences in AEP for 3 or more subjects were also found in temporal pole (TP), angular gyrus, IFG, gyrus rectus, and orbitofrontal cortex (OFC). Furthermore, the clustering algorithm identified AEP differences to the control stimulus in few electrodes targeting areas MFG, transverse frontopolar gyrus, parahippocampal gyrus, and fusiform gyrus.

**Figure 3.**
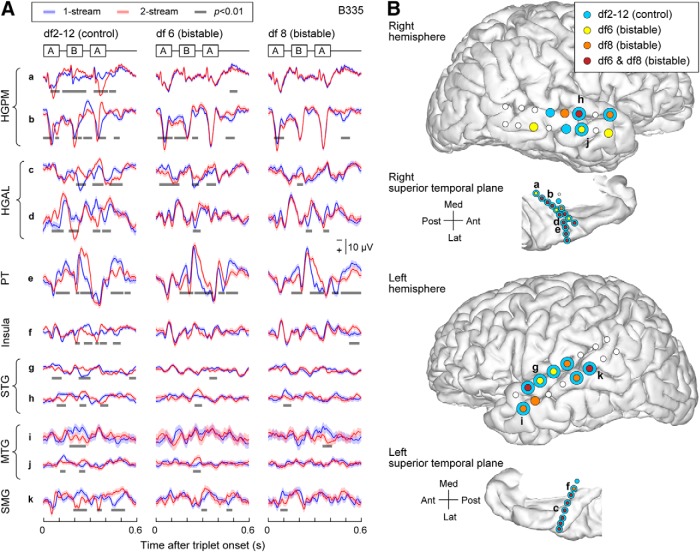
Bilateral electrode coverage and spatial neural response map for Subject B335. ***A***, Differences in AEPs over trials in 1-stream (blue) and 2-stream (red) were identified as clusters of time points at *p* value below significance level 0.01 according to the permutation-based cluster test and FDR correction (gray). Exemplar AEPs from several cortical areas of interest were shown for the control (df2-12; left column) and the bistable (df6/df8; center/right columns) stimuli. At the control stimulus, percept-related AEP differences are confounded with stimulus-related differences. ***B***, Spatial maps were derived based upon statistical analysis of AEPs in the time domain. Differences in percept-related AEPs were identified in several temporal and frontal-parietal areas for the control stimulus (df2-12; in light blue) and the bistable stimuli (df6 in yellow; df8 in orange; sites selected at both df6 and df8 were plotted in maroon). Only the sites on the hemispheric convexity and in the superior temporal plane were plotted here (for the complete list of sites that exhibited significant AEP differences, see Table 2). Concentric circles represent the contacts showing AEP differences at both control and bistable conditions.

**Figure 4.**
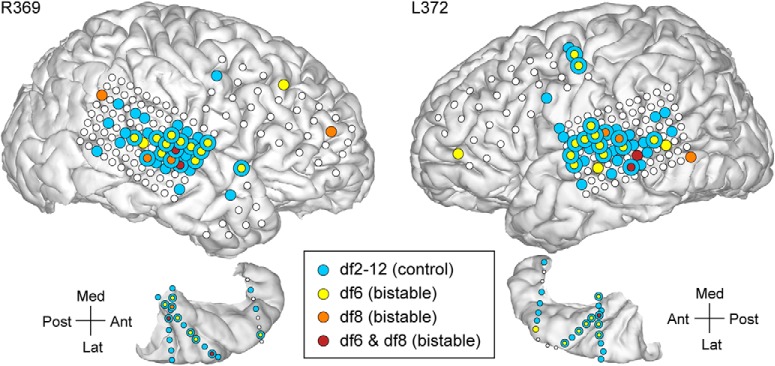
Electrode brain coverage and spatial neural response map for Subject R369 (left) and Subject L372 (right). Only the contacts on the hemispheric convexity and in the superior temporal plane were shown here (for the complete list of sites that exhibited significant AEP differences, see Table 2). Recording sites that exhibited significant differences in percept-related AEPs in the control (light blue) and bistable conditions (yellow/orange; maroon for overlap). Concentric circles represent sites where AEP differences were found at both control and bistable stimuli.

The majority of significant clusters (one or more per contact) were associated with areas HGPM, HGAL, PT, STG, MTG, SMG, insula, PoCG, and PreCG. Exemplar AEPs and related clusters at contacts in several cortical areas of interest are illustrated in Figure 3*A* for Subject B335. Extensive coverage of the lateral surface by subdural grid arrays revealed AEP differences between 1-stream and 2-stream at numerous sites in STG and MTG on both the right and the left hemispheres (see R369 and L372; Fig. 4). Subject B335 had extensive coverage of AC and surrounding auditory-related temporoparietal cortex in both hemispheres (Table 2). Other ECoG subjects had electrodes placed over PreCG, MFG, IFG, gyrus rectus, and OFC; and some of those showed significant differences in the statistical analysis (Table 2).

The statistical significance of all identified clusters was used to construct a spatial map of neural response to dissimilarities between 1-stream and 2-stream (Figs. 3*B*, 4). The smallest FDR-corrected site *p* value per given experimental block was deemed to represent the score of the respective site. The spatial map included all recording sites that had their score of statistical significance. All *p* values associated with the sites of the spatial map took values <0.01 after the FDR correction.

### Neural responses to bistable stimuli

Studying neural correlates of perception with stimulus df2-12 provided the advantage of relying on good accuracy of perceptual data. That was because the triplet-based trials were split into 1-stream and 2-stream according to the subjects' behavior but were also vetted by direct comparison with stimulus features. At df2-12, perceptual switching events were externally manipulated by changes in stimulus property. The changes in df separation were extreme, from 2 to 12 semitones and back, promoting almost exclusively integration or segregation (van Noorden, 1975). This approach has a major limitation: the distinct perceptual organizations were created using different stimuli. Therefore, the changes observed in neural activity may have reflected differences between the perceptual states but also physical differences of stimuli per se. To avoid this problem and focus only on neural correlates to perception, bistable auditory stimuli were further investigated. In doing so, however, we were compelled to rely exclusively on the subjects' self-reported perception and their individual assessment of stimulus ambiguity.

The ECoG data recorded during the bistable conditions df6 and df8 were included in the same nonparametric cluster-based statistical test as block df2-12. Significant differences between AEPs associated with 1-stream and 2-stream trials were identified. They occurred in the same cortical areas reported for df2-12 but had a more restricted spatial distribution and, overall, showed a more subtle clustering effect (*p* < 0.001, χ^2^ test). Specifically, clusters of statistical significance in the bistable conditions df6 and df8 were distributed across 36 (38) recording sites in Subject B335, 1 (2) sites in L357, 31 (10) sites in R369, 31 (7) sites in L372, 1 (3) sites in R376, 15 (16) in R399, 1 (2) in L409, and 21 in R413, respectively (Table 2). The score of percept-related differences was calculated for each site, and the spatial map representing all sites of statistically significant score was drawn (e.g., Figs. 3*B*, 4). Overlaps between the spatial maps obtained for the control and bistable stimuli were observed in core (HGPM) and non-core AC (HGAL, PT, PP, STG) as well as in other temporal (MTG, ITG, STS, TP), parietal (SMG), and frontal (IFG, MFG, OFC) regions (Figs. 3*B*, 4, concentric circles). For a summary of results at group level, see also Figure 1*B* and Table 2.

When compared per individual site and percept type, temporal activation in AC and in temporoparietal auditory-related cortex shared strong similarities across the control and bistable conditions (Fig. 3*A*). In contrast, temporal patterns were different across multiple areas of interest. Specifically, AEPs in the auditory core cortex (Fig. 3*A*; HGPM, sites a, b) were characterized by short latencies and large amplitude, and they maintained a robust isomorphic representation of acoustic stimulus features, including onsets and offsets of individual tones within the triplet. Non-core auditory cortical areas, such as HGAL (sites c, d) and lateral STG, MTG, SMG (sites g-k), as well as insula (site f), were characterized by longer latency responses and broader AEP peaks and appeared to represent the stimuli in a more abstracted form. AEP waveforms at sites in PT were found to resemble the shape of the responses from the auditory core cortex (site e).

**Figure 5.**
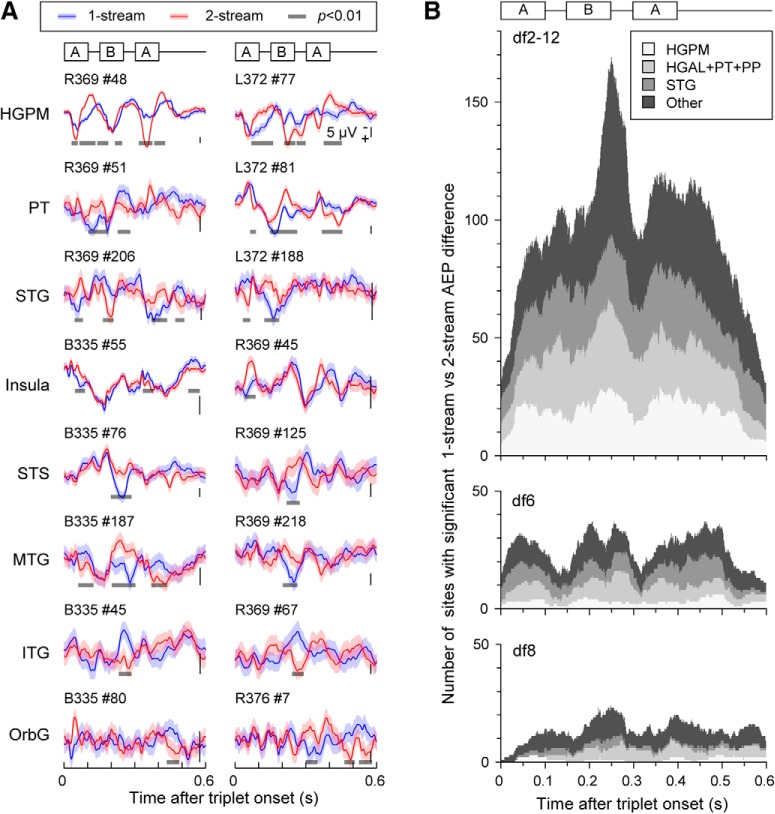
***A***, AEPs from several cortical areas of interest showed qualitative similarities of waveforms across different subjects (all AEPs were shown at df2-12; for acronyms of cortical areas, see Table 2). Vertical bar represents magnitude of AEPs varied across subjects. ***B***, All significant clusters of differences between AEPs of 1-stream and 2-stream percepts were tallied at each time point of the 600 ms trial, separately for the core AC (HGPM; in white), non-core AC in the superior temporal plane (HGAL, PT, PP; in light gray), non-core AC STG (in dark gray), and the rest of cortical areas (Other; in black). The sum was taken across all contacts and all ECoG subjects, separately for the control (df2-12, top) and bistable (df6, middle panel; and df8, bottom) stimuli. Tally of significant clusters showed local maxima around the position of tones in triplet *ABA*_ with maximal overlap after tone *B* onset.

Recording sites in HGPM, HGAL, PT, STG, and MTG showed significant clusters around the amplitude peaks of the response to tone *B*, with a maximum difference between 1-stream and 2-stream at 60–130 ms after tone *B* onset (Fig. 3*A*; for additional examples, see Fig. 5*A*). Such clusters were identified in exemplar Subject B335 at ∼60–80 ms after the onset of tone *B* in HGPM (Fig. 3*A*, sites a, b), 90–110 ms in HGAL (sites c, d), 100–130 ms in PT and STG (sites e, g, h), and 80–100 ms in MTG (sites i, j). A significant cluster with much longer latency was found in HGPM, HGAL, PT, and in some contacts from STG and MTG with a maximum difference between 1-stream and 2-stream at 50–90 ms after second tone *A* onset (Figs. 3*A*, 5*A*). Our findings were consistent with results from previous human noninvasive functional neuroimaging studies using similar stimuli (Gutschalk et al., 2005; Snyder et al., 2006; Hill et al., 2012).

Significant clusters at similar latencies after tone *B* onset were also detected at sites in cortical areas STS, SMG, insula, and ITG. Likewise, similar latencies after second tone *A* onset were found at additional sites in the temporal (insula), parietal (SMG), and OFC cortex.

Maximum difference between AEPs computed over 1-stream and 2-stream trials, at both tone *B* and second tone *A* clusters, varied between 4 μV (insula, STG, MTG) to 18 μV (HGPM, PT) at an average of 7–12 μV (HGPM, HGAL but also sites in STG, MTG, OFC) depending on the subject. Examples of AEP waveforms for Subject B335 (at blocks df2-12, df6, df8) and for Subjects R369, L372, R376 (at block df2-12) are illustrated in Figures 3*A* and 5*A*. The amplitudes of AEPs at contacts outside the auditory and auditory-related cortex were smaller, in general. This was not surprising, given that the neural activity in those areas was not typically locked to the auditory stimulus. Under these circumstances, it was difficult to assess whether the reduced amplitude of AEPs had reflected a lack of response to stimulus or whether it had just been an inherent consequence of the averaging process over trials (triplets) less well aligned due to the increased time jitter. Nevertheless, we found that clusters of significant difference between 1-stream and 2-stream percepts occurred in areas outside the AC quite consistently at tone *B* as well: both in bistable and (in much larger number) in control conditions (Fig. 5*B*).

Our data supported the hypothesis advanced by Hill et al. (2012) that percept-driven neural activity is rather linked to the relative position of tones within the sequence than to the stimulus low-level properties. Indeed, significant clusters were found at similar times at contacts placed in core (HGPM) and non-core (HGAL, PT, PP and STG) AC, with maximal overlap during tones *A*, *B* and second *A* (0–100, 200–300, and 350–400 ms after triplet onset) and with an additional overlap at ∼400–500 ms during the silent interval (Fig. 5*B*); tally shows the number of significant clusters at each time *t* in HGPM (in white), HGAL, PT, PP (light gray), and STG (dark gray). At sites outside the AC, significant clusters overlapped predominantly at tone *B* (Fig. 5*B*, black). This result was consistent across subjects and blocks, although the number of cluster points showing differences between 1-stream and 2-stream trials at tone *B* was especially very high during the control condition. In the latter case, the observed differences could have been driven by the dynamic changes in the stimulus properties at df2-12 rather than being influenced by perception. However, during block df2-12, frequency *f_B_* was kept fixed at *f_B_* = 1000 Hz, whereas frequency *f_A_* was either at 2 or at 12 semitones above *f_B_*. If differences in AEPs were to occur because of such large changes in *f_A_*, one would expect to identify them around the *A* tones instead of *B*, which was not the case (Fig. 5*B*, top).

Some contacts showed significant clusters in the 60–130 ms time window after tone *B* onset with opposite sign for the difference AEP_2_(*t*) − AEP_1_(*t*) when compared across conditions (here AEP_1_ and AEP_2_ were the AEPs calculated over 1-stream and 2-stream trials). Such example is site d for Subject B335 (Fig. 3*A*; second cluster at df2-12 vs first cluster at df6 and df8). This indicated a larger positive peak when the segregated (2-stream) rather than the integrated (1-stream) percept was reported at df2-12, and the opposite for df6 and df8. The clusters, however, did not overlap; the cluster at df2-12 occurred earlier at ∼60 ms after tone *B* onset, whereas the clusters at df6, df8 were found at ∼105 ms after tone *B* onset. This result was consistent with studies based on MEG and EEG recordings (Gutschalk et al., 2005; Hill et al., 2012) that reported a positive peak difference between 2-stream and 1-stream percepts at ∼74 ms followed by a negative peak at ∼110 ms after tone *B* onset. Given that the position of depth electrodes in relation to gray matter might affect their polarity, we refrained from averaging the difference waveforms AEP_2_(*t*) − AEP_1_(*t*) across sites. Instead, we counted how many significant clusters of difference between AEP_1_ and AEP_2_ were found at each time point during the trial for all ECoG subjects and all contacts. This tally revealed two peaks at ∼60 and 110 ms after tone *B* onset at df6, and a broader peak encompassing those time windows at df8 (Fig. 5*B*).

### Classification

**Figure 6.**
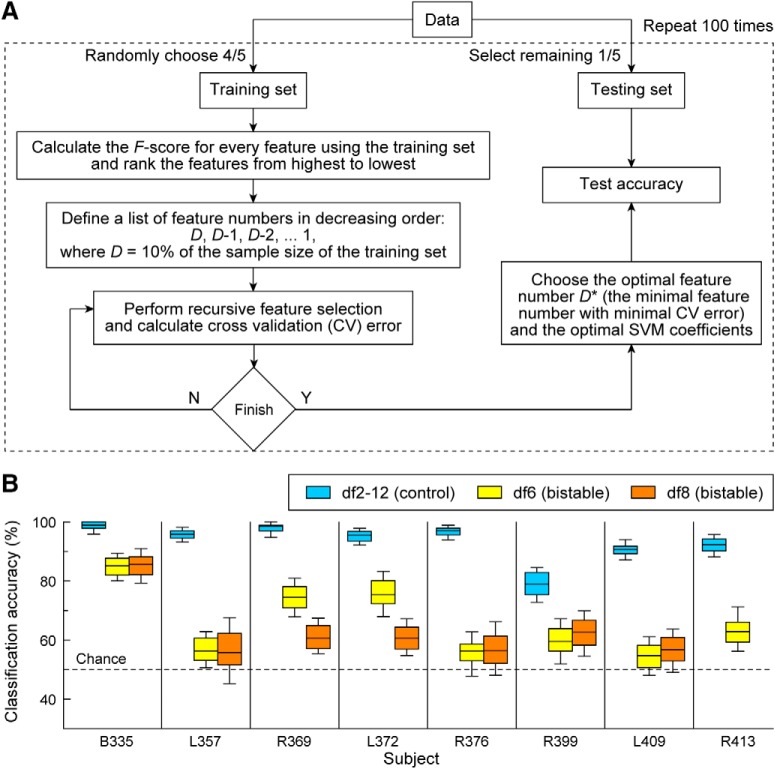
Percept classification. ***A***, Numerical scheme of the classifier. Recursive feature selection and SVM algorithms were run multiple times to construct the distribution of accuracy values and to identify the set of optimal features that contribute to the classification. ***B***, For each subject, distributions of test accuracies were computed from 100 iterations of the classifier applied to the control (light blue) and bistable (yellow/orange) stimuli.

We corroborated our results obtained by nonparametric statistical methods with the outcome of a classifier that combined SVM with a recursive feature selection procedure (Fig. 6*A*). The classifier was defined by several iterative steps to address the peculiarities of the ECoG data (see Materials and Methods) and to ensure independence of the training and testing datasets. It yielded a distribution of accuracies that was used to estimate the mean and median test accuracy (Fig. 6*B*). The classifier showed very good performance for separation of trials in the control condition with median and mean accuracies of 98%, 95%, 97%, 95%, 96%, 79%, 90%, and 92%. Classification of trials in the bistable condition was also achieved at above chance levels in all subjects (*p* < 0.001 for all 8 subjects; one-tailed *t* test run over the set of 100 accuracy measurements compared with chance level 0.5). However, the classification accuracy of bistable stimuli was good (74%–85%) in 3 of 8 but rather weak (55%–63%) in 5 of 8 ECoG subjects.

**Figure 7.**
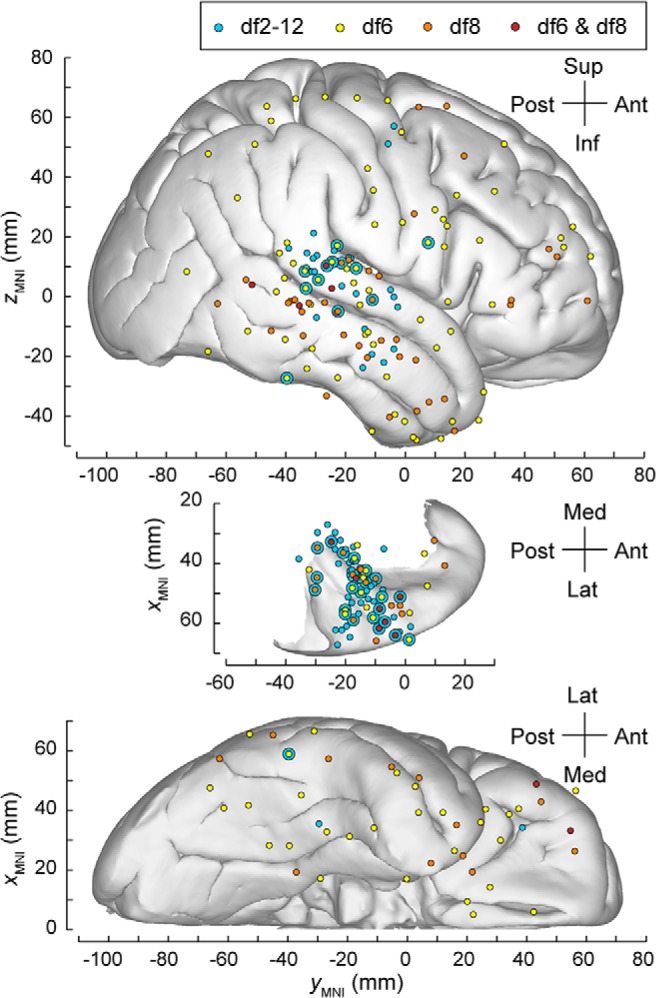
Classifier-driven ECoG group spatial maps were obtained from the analysis performed in Figure 6. Differences in spatially distributed LFP signals were detected by selection of optimal feature sets. The corresponding spatial maps for control (light blue) and bistable stimuli (df6 yellow; df8 orange; overlap in maroon) were defined by all recording sites selected as relevant optimal features through the classification process. Then, they were projected on the lateral, superior temporal, and ventral views. ITG sites were plotted in both the lateral and ventral view.

A spatial map was created using all recording sites that belonged to the optimally selected feature set. The classifier-driven spatial maps of percept-related AEP differences derived for the control and the bistable stimuli overlapped (Fig. 7). They were also found to be consistent with many of the recording sites that were selected for significant difference by the univariate analysis in either control (data not shown; but for the areas selected for control, see Table 2) or bistable (Fig. 1*B*) conditions.

### Cluster-based analysis of the envelope of high gamma ERBP

High gamma power was reported by several studies (Nourski et al., 2013, 2014a,b) to carry information about task-related neural activity, and it is less sensitive to volume conduction than low-frequency AEPs. A quantitative analysis of the ERBP envelopes in the high gamma frequency band was performed for all recording sites and the control and bistable conditions in each ECoG subject. Clusters at uncorrected *p* value <0.05 were identified for all subjects in cortical areas reported for AEPs (Table 2), in the control and both bistable conditions for overall 138, 98, 77 of 1519 number of sites, in HGPM, HGAL, PT, PP, STG, MTG, ITG, STS, INS, TP, PHG, SMG, ANG, PoCG, PreCG, TFP, SFG, MFG, IFG, and OFC. However, none of these clusters remained significant after the FDR correction. Negative results of the analysis of high gamma power compared with the analysis of the LFP signal are consistent with earlier reports about the differences in classifier performance based on these two measures of cortical activity (Nourski et al., 2015). In particular, LFPs rather than high gamma activity measured by intracranial recordings provided better classification accuracy of speech consonants.

**Figure 8.**
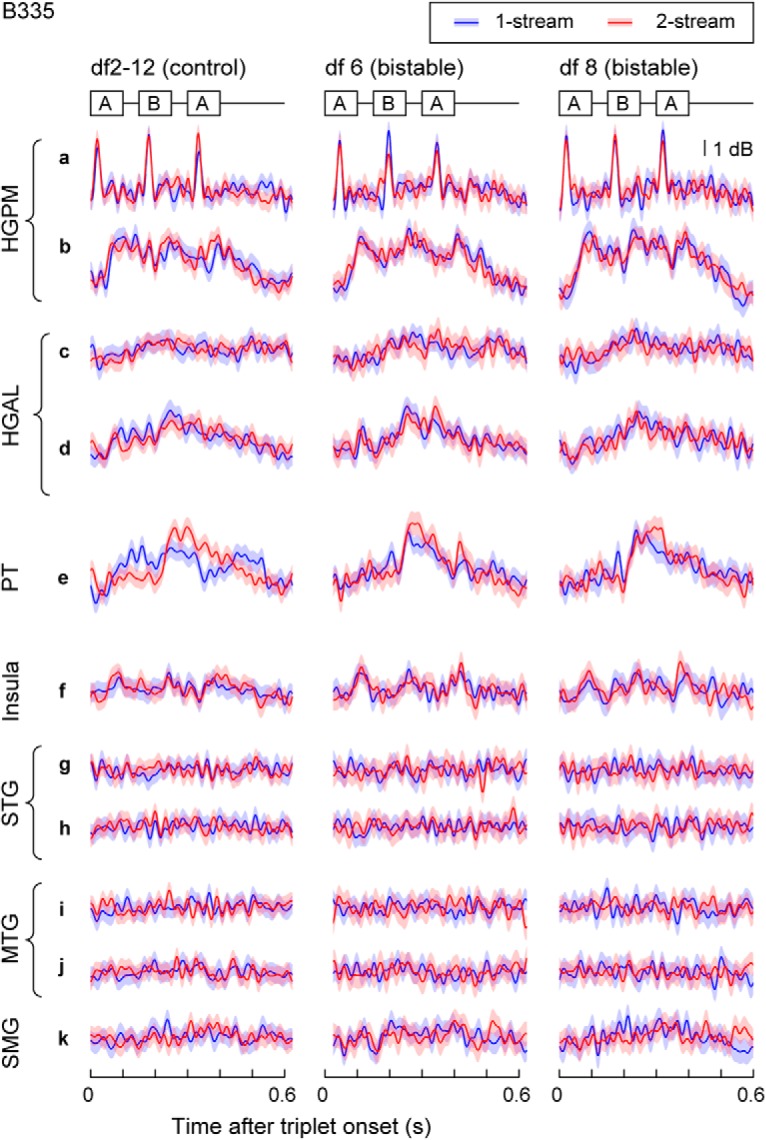
Exemplar high gamma band (70–150 Hz) responses to the control (df2-12; left column) and the bistable (df6 and df8; middle and right columns) for Subject B335. Same sites as in Figure 3*A* were shown. Differences in the mean ERBP over trials in 1-stream (blue) and 2-stream (red) were analyzed with the permutation-based cluster test. None of the identified clusters survived the FDR correction step.

Exemplar high gamma ERBP envelopes are shown for Subject B335 in Figure 8. They are presented for the same sites as those that exemplified AEP univariate analysis (Fig. 3*A*). ERBP at HGPM (site a) resembled the profiles of firing rates obtained from single-unit recordings in area A1 of nonhuman primates during streaming of triplets (Micheyl et al., 2005), in support to the hypothesis that high gamma activity might be correlated with neuronal firing. In Micheyl et al. (2005), measurements were taken only from neurons with the best frequency the same of tone *A*, and found that amplitudes of responses to the *B* tone decreased with larger df, but the macro contact presumably recorded neural activity from a population of neurons spanning a wider range of best frequencies, so a direct comparison between these datasets was limited to qualitative comparisons (e.g., waveform of the response) rather than quantitative estimates of magnitudes.

### Activation of AC reveals differences between 1-stream and 2-stream during maintenance of percepts and at transition between them

A group-level statistical analysis was performed to test the hypothesis that common neural substrates supported maintenance of bistable percepts and perceptual switches in auditory streaming of triplets. This analysis was restricted to the AC ROIs (HGPM, HGAL, PT, PP, STG) as they exhibited the most consistent activation across control and bistable conditions. The group-level statistics were done independently from the univariate and multivariate statistics described in previous sections.

**Figure 9.**
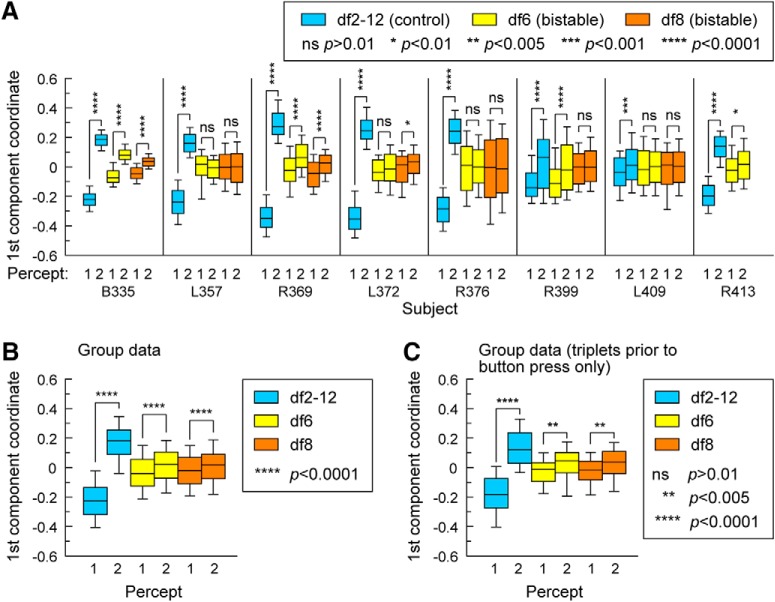
Group-level statistics. For each ECoG subject, single-trial LFPs from all contacts placed in the AC are associated with a point (the first component coordinate) in a one-dimensional space by a diffusion map. ***A***, Differences between 1-stream and 2-stream trials during the maintenance of percepts are evaluated by Wilcoxon rank-sum test applied to their first component coordinates. This is done for control (df2-12, blue) and bistable (df6, yellow; df8, orange) conditions and for each subject separately. Plots include the 10th, 25th, 50th (median), 75th, and 90th quantiles. Differences are statistically significant for a *p* value <0.01 after the FDR correction. ***B***, Same as in ***A***, but with statistics performed on the group data of projections of 1-stream trials and 2-stream trials from all 8 ECoG subjects. ***C***, Differences between trials before the switch between percepts are evaluated by Wilcoxon rank-sum test applied to their projections. Switch into 1-stream is labeled “1”; switch into 2-stream is labeled “2.” Trials from all ECoG subjects were combined for the purpose of the analysis.

For each ECoG subject and experimental block, single-trial LFPs from all sites in the AC were mapped onto a one-dimensional embedding space. Then the projections of individual trials (also called first component coordinates) were used in the analysis as proxy for the multidimensional data (Coifman and Lafon, 2006; Mishne et al., 2016). Our analysis was motivated by recent theoretical results (Nadler et al., 2006) that found that first component coordinates of diffusion maps were well suited to uncover bistable properties of large-scale spatiotemporal data. Wilcoxon rank-sum tests were performed between projections of 1-stream and 2-stream trials during sustained perceptual states for each subject and condition separately (Fig. 9*A*) and for the group data across all 8 ECoG subjects (Fig. 9*B*). Wilcoxon rank-sum test was also performed between projections of the trials immediately before the perceptual switches from 1-stream to 2-stream and vice versa over group ECoG data (Fig. 9*C*). The analysis showed better separation of percepts during the control condition for individual subjects and for the ECoG group (Fig. 9, boxplots in blue), potentially due to encoding by AC of stimulus-related differences (Fishman et al., 2001; Micheyl et al., 2005) in addition to percept-driven changes. However, separation of percepts was also found during presentation of invariable bistable stimuli (Fig. 9, boxplots in yellow or orange). Activation of core AC rather than non-core AC appeared to contribute more to the classification of percepts during block df2-12 as Subjects R399 and L409 showed the lowest level of separation between 1-stream and 2-stream while they also had the most reduced coverage of HGPM (3 sites in R399, 1 in L409; see Table 2). On the other hand, activation of non-core AC rather than core AC was found to play a bigger role in identifying differences between percepts during blocks df6 and df8. Subjects L357 and L409 did not show significant perceptual differences during bistable conditions, but they also had the most reduced coverage of areas HGAL, PP, PT, and STG compared with others (Table 2). This result mirrors findings from the univariate analysis where L357, L409 (and R376) had the lowest number of sites showing significant differences during bistable conditions (Table 2) as well as findings from the multivariate analysis where data from same subjects showed the lowest accuracy of percept classification (Fig. 6*B*). At the group level, however, differences between trials compared during the maintenance of percepts and at the switches between them were found to be statistically significant at *p* values <0.005 (Fig. 9*B*,*C*). This result supports the hypothesis that AC plays a key role in the encoding of dynamic features of stable percepts as well as in the encoding of transitions between them in auditory streaming of triplets.

## Discussion

We investigated the localization and dynamic properties of neural correlates of auditory streaming of triplets. Most experiments in humans had used similar stimuli with noninvasive recordings with limited spatial resolution (EEG, MEG) or limited temporal resolution (fMRI). LFPs and single-unit and multiunit recordings in animals identified temporal details of the neural response to acoustic stimuli but focused on specific brain areas and did not take perception into account. Our study bridges these disparate aspects of analysis by obtaining concurrent temporal and spatial high-resolution ECoG recordings from subjects actively performing the task. The focus was on defining spatial brain response maps to auditory bistable perception. However, their construction relied heavily on temporal features of the LFP signal at each recording site and comparisons over time windows with resolution as low as 20 ms. We thereby uncovered a spatially distributed cortical map of AEP differences between 1-stream and 2-stream percepts that simultaneously provides fine temporal characterization of the percept-related trials.

### Control versus bistable stimuli

Statistically significant and classifier-based AEP differences between percepts were identified in core and non-core AC, surrounding auditory-related temporoparietal cortex, and frontal areas. The spatial response maps in the control and bistable conditions largely overlapped, but the bistable maps were sparser. The salience of the perceptual switches, which were more pronounced for the control than for bistable stimuli, possibly caused the stronger perceptual effect observed during control condition. The underlying distinct, low-level acoustic stimulus features might be another reason, but those should have a stronger impact on the AC rather than areas, such as frontal cortex.

### Cortical areas activated during bistable perception

Several cortical regions identified by our statistical analysis have been reported by studies of bistable perception using stimuli other than streaming of triplets. Right IFG and parietal cortex were shown to be active during visual perceptual alternations (Knapen et al., 2011). fMRI studies on verbal transformations showed activation of left IFG, AC, STG, SMG, and insula (Kondo and Kashino, 2007). For streaming of triplets, bilateral fMRI activation to perceptual switches was reported in insula, AC, and SMG (Kondo and Kashino, 2009). These previous studies focused on the events time-locked to the perceptual switching rather than the dynamics of the perceptual states per se. In contrast, our analysis was done over the maintenance phase of the percepts while ignoring the switches. To test whether switches as well as maintenance of the percepts could be regulated by common neural substrates, we performed a group-level analysis on the recordings obtained from all sites in AC. We found that activity in AC encoded differences between 1-stream and 2-stream percepts and also discriminated between transitions from one percept to another.

### Classification of competing auditory percepts

A relatively weak classification performance for bistable auditory streaming of triplets was recently reported for MEG recordings (Billig et al., 2018; Sanders et al., 2018). Those studies also found accuracies <60%, despite comparing three different classifiers and using a larger pool of subjects and more experimental blocks. Our data-driven classifier used invasive brain recordings and showed considerably stronger classification performance in the control than the bistable condition. However, the percepts were much more clearly defined, and the switch between them was salient in the former case. This result is consistent with other studies of bistable perception that probed the effect of stability of the perceptual states on the subjective response. The difference is that we used stimuli that stabilized unstable competing percepts as opposed to using attentional cues to disrupt stable percepts (Intaite et al., 2014). It remains an open question whether the strength of percept stability and the classification accuracy are, indeed, directly related.

### ECoG subjects reported longer percept durations than healthy subjects

These results were possibly due to anticonvulsant epileptic drug-related changes in ECoG subjects' brain excitation-inhibition balance and fit well with the theories for bistable perception. Competition models showed that mutual inhibition together with slow negative feedback, such as adaptation, or noise, could produce switching (Laing and Chow, 2002; Curtu et al., 2008). In particular, an increase in inhibition made it harder for the switches to occur, potentially leading to winner-take-all responses (Shpiro et al., 2007; Curtu and Rubin, 2011). The results are also supported by recent studies on auditory streaming of triplets that found correlation between alternating patterns, glutamate concentrations in AC as well as GABA concentrations in IFG (Kondo et al., 2017).

### Pitfalls and caveats of the analysis

The electrodes used for implantation in this study had small diameter; and presumably, they recorded activity generated in the immediate vicinity of the recording contact (i.e., the LFP). However, intracranial recordings can reflect neural activity generated locally and remotely, due to volume conduction (Buzsáki et al., 2012). Several factors precluded the localization of LFP generators and the interpretation of LFP characteristics. Methods, such as amplitude spatial gradients, could not be used to identify local generators as they are not suited for the analysis of recordings made at sites distributed across several gyri or for oblique penetrations through highly localized fields (Tenke and Kayser, 2012); the geometry of the current sources, which strongly affects LFP amplitude, was unknown; then the polarity of depth electrode recordings might not be reliably determined because one could never be certain which side of the dipole is on. We acknowledge that the spatial maps reported herein, while providing important insights into the cortical representation of AEP differences between percepts, should not be taken as proof of origin for sources generating them.

### Comparison with other studies of auditory streaming of triplets

Single-unit recordings from AC and cochlear nucleus in animals (Fishman et al., 2001; Micheyl et al., 2005; Pressnitzer et al., 2008) were obtained during presentation of repeating-triplet sequences known to cause streaming in humans. The recordings were made from neurons with best frequency at tone *A* while systematically varying df, and showed suppression of the spiking activity during tone *B* for larger df (Micheyl et al., 2005). This was hypothesized to be indicative of stream segregation. However, the approach was limited in scope due to the use of different stimuli to create distinct perceptual organizations. High gamma power waveforms at certain HGPM sites in this study were similar to firing rates profiles reported in primates but showed no significant difference between percepts, possibly due to sampling of more heterogeneous local neuronal populations.

Noninvasive recordings in humans found neural correlates of persistent perceptual states in streaming of triplets in AC and the intraparietal sulcus (Cusack, 2005; Gutschalk et al., 2005; Snyder et al., 2006; Hill et al., 2011; Billig et al., 2018). Percept-related MEG responses were localized to AC, occurring ∼60 ms after the onset of tone *B* and had larger amplitudes at the 2-stream percept (Gutschalk et al., 2005). In contrast, EEG recordings showed perceptual effects in AC at longer latencies, at time intervals overlapping the second tone *A* in the triplet (Snyder et al., 2006). However, that study used stimulus modifications not stimulus bistability to induce changes in perceptual state. Hill et al. (2012) disentangled the effects due to changes in the stimulus parameters from those due solely to perception. In EEG recordings, they showed the former to occur at latencies as reported by Snyder et al. (2006), and the latter at latencies reported by Gutschalk et al. (2005). Our data confirm and expand those findings. Clusters of significant difference between 1-stream and 2-stream were identified within the triplet epoch at times comparable with those observed in MEG and EEG studies, in non-core auditory cortical areas HGAL, PT, and STG, but also in HGPM and MTG. Moreover, ECoG recordings from these cortical areas help explain the topographic dissimilarities of the triplet epoch waveform between MEG (Gutschalk et al., 2005) and EEG data (Hill et al., 2012). MEG measures primarily the activity of pyramidal cells situated perpendicular to the cortical surface. Scalp EEG also detects tangential activity from the depth, but it is most sensitive to radial sources. It is then possible that MEG and EEG signals are best approximated by intracranial recordings at depth and subdural electrodes, respectively. We found, indeed, that AEPs in HGPM resemble the MEG waveforms reported by Gutschalk et al. (2005), and that AEPs at sites in STG and MTG resemble the EEG waveforms reported by Hill et al. (2012) (Fig. 3).

To our knowledge, our study is the first to report ECoG differences between 1-stream and 2-stream percepts in auditory streaming. An early attempt for such characterization was not successful despite evidence for correlates of frequency separation that was widespread (Dykstra et al., 2011). However, that study focused on much shorter durations of 6.5–10 s, whereas we used 5-min-long stimuli to analyze perceptual alternating states. It is known from behavioral studies that the probability of stream segregation builds up on the scale of several seconds after the stimulus onset, and that typically, the first percept has longer durations than the subsequent ones (Pressnitzer and Hupé, 2006). Therefore, our findings, as opposed to those in Dykstra et al. (2011), might have been drawn from a distinct phase of bistable perception: the stable alternation cycle versus the buildup.
